# Clinical management and burden of cytomegalovirus in D+/R-Kidney transplant recipients in Canada

**DOI:** 10.3389/fimmu.2025.1618748

**Published:** 2025-09-18

**Authors:** John Gill, Andrew A. House, Zain Chagla, Jean Tchervenkov, S. Joseph Kim, Amanda Vinson, Carlos Cervera, Paul A. Keown, Sonia Lai Wing Sun, Christina Khoury, Christiane Ghakis

**Affiliations:** ^1^ Department of Medicine, University of British Columbia, Vancouver, BC, Canada; ^2^ Department of Medicine, Western University, London, ON, Canada; ^3^ Department of Medicine, McMaster University, Hamilton, ON, Canada; ^4^ Department of Surgery, McGill University, Montreal, QC, Canada; ^5^ Department of Medicine, University of Toronto, Toronto, ON, Canada; ^6^ Department of Medicine, Dalhousie University, Halifax, NS, Canada; ^7^ Department of Medicine, University of Alberta, Edmonton, AB, Canada; ^8^ Medical Affairs, Merck Canada Inc, Kirkland, QC, Canada

**Keywords:** cytomegalovirus, kidney transplantation, antiviral prophylaxis and treatment, immunosuppression, superinfection, leukopenia, hospitalization, graft failure

## Abstract

**Purpose:**

To document prophylactic practices, infection patterns, and disease burden to inform strategies for CMV management in high-risk kidney transplant recipients.

**Methods:**

A retrospective cohort of 311 consecutive CMV D+/R- kidney recipients were enrolled from 7 Canadian programs over 4 years (2018-2021) to provide data on demographic, clinical, therapeutic and health resource use during the 1st year post-transplant.

**Results:**

Themedian age was 58 (46, 67) years, 69% were male, and 53% were White. Diabetes was the principal cause of kidney failure (19%). 208 (69%) received a deceased donor graft; 76 (24%) had ATG induction, and 84% had maintenance therapy with tacrolimus and MMF/MPA ± prednisone. All received antiviral prophylaxis, 90% with valganciclovir, for a median of 180 days. 106 (34%) developed CMV viremia (median peak viral load 14,224 IU/ml) at a median of 218 days, of whom 46 (43%) had CMV disease and 15 (14%) had recurrent infection. Myelotoxicity occurred in 121 (39%) patients at a median of 88 days, lasting a median of 30 days. Opportunistic infections occurred in 119 patients (38%) at a median of 53 days. 141 patients (45%) were hospitalized, 50 (16%) more than once. 20 patients (6%) had biopsy-confirmed rejection, and 293 (94%) were alive with a functioning graft at 1 year.

**Conclusion:**

Current prophylaxis strategies fail to prevent CMV infection in 34% of high-risk patients. Myelotoxicity, opportunistic infection, reduced immunosuppression, and hospitalization remain common and serious complications. More effective and less toxic personalized treatment strategies are required to minimize these risks and burdens.

## Introduction

Cytomegalovirus (CMV) infection is a common complication of kidney transplantation which remains a major therapeutic challenge with both clinical and economic implications (1, 2). The risk of CMV infection is highest in immunologically naïve seronegative recipients (R-) of organs from a seropositive donor (D+), comprising approximately 10% to 20% of kidney transplants (3–5). Anti-viral prophylaxis has reduced and delayed the risk of CMV infection in these patients (5–9), which occurs in 10 - 50% (3, 10–12) of subjects throughout the first post-transplant year (1).

CMV infection may present with a broad range of disease expression from asymptomatic viremia to CMV syndrome or end-organ disease including esophagitis, enteritis, pneumonitis, encephalitis, pancreatitis, and other target organ damage (8). It may occur alone, or in a complex context of multiple disease events. For example, CMV is often diagnosed coincident with other bacterial, viral or fungal opportunistic infections in the post-transplant setting, complicating diagnosis, and management (13–15). The occurrence of CMV infection and graft rejection are also closely correlated, and models of combinatorial risk have been proposed (16). However, it remains unclear whether the immune modulating effect of the virus enhances graft rejection, or treatment of rejection increases the risk of viremia (8, 17).

Despite overall reduction in the incidence, severity, and consequences of CMV infection, the management of D+/R- patients remains challenging. CMV viremia and disease occur more commonly within this group and may result in serious complications, despite current prophylaxis strategies (1, 5, 18, 19). Prolonged prophylaxis can itself have important costs and toxicity resulting in serious clinical leukopenia (20) which may in turn lead to co-infection or secondary reduction in immune suppression resulting in breakthrough rejection.

Measurement of CMV viral load is considered the most relevant index to document viral replication, to diagnose infection, and to determine treatment effect. It has also been included as a surrogate marker in clinical trial settings (1, 7, 8, 21). Accurate monitoring of viral load and the use of anti-viral prophylaxis or pre-emptive therapy have mitigated the lethal consequences of CMV infection and diminished the early indirect consequences and costs of care (1, 8). Calculation of viral load kinetics has been proposed to guide the frequency of sample measurement and to facilitate the effective use of therapy (22, 23), but this has not yet been incorporated into clinical practice.

Despite the availability of US, European and international clinical guidelines, we lack current Canadian published data on the clinical treatment and burden of CMV infection as we optimize management strategies for preventing infection in this high-risk kidney transplant population (8, 24–26). The recommendations of the Canadian Society of Transplantation CMV Consensus Working Group were published almost two decades ago (27) and a more recent single-center Canadian pediatric study (28) does not provide evidence that is applicable to the broader landscape of transplantation. Consequently, viral testing, prophylaxis or pre-emptive therapies vary between transplant programs.

The goal of this study was to complement existing knowledge in both live donor (LD) and deceased donor (DD) D+/R- patients who were at highest risk for CMV infection and disease by providing a comprehensive understanding of current practice, to understand disease burden, provide precise and current data for economic modeling, and to highlight opportunities to improve therapy and healthcare resource utilization in this patient population. We report the dynamics and cumulative frequencies of the key outcomes of interest which will enable us to model the interplay of these events and identify groups with differential disease burden to guide therapy.

## Methods

### Objectives

The primary objectives of this study were to describe: (a) the incidence of CMV infections (including but not limited to: reactivations, number of recurrences as well as incidence of resistant or refractory infection or disease); (b) incidence of CMV antiviral-related myelotoxicities, including leukopenia and neutropenia, and their impact on patient’s treatment adjustments (decrease/discontinuation of immunosuppressants and CMV antiviral agents); and (c) the association of CMV management with health care resource utilization (HCRU), including the length of hospital stay (LOS) and frequency of hospital admissions.

### Study design

This retrospective, consecutive subject, multicenter cohort study was designed to examine the clinical and economic burden and unmet prophylactic needs in CMV seronegative adult recipients (R-) 18 years or older of a kidney transplant from a CMV seropositive donor (D+). Recipients who participated in an interventional trial in the prior 90 days, had received another organ or stem-cell transplant, or were exposure to letermovir were excluded. The study was conducted in seven major transplant centers in Canada (**Table 1**) and included consecutive CMV seronegative recipients in each participating transplant program who received a kidney transplant from a CMV seropositive donor from January 1st, 2018 to December 31st, 2021, and for whom continuous follow-up data were available. A pragmatic sample of approximately 300 CMV D+/R- kidney transplant recipients was considered adequate to provide preliminary evidence of CMV treatment and burden.

**Table 1:** Patient demographics and baseline characteristics.

	Living donor (N=103)	Deceased donor (N=208)	Overall (N=311)
Recipient sex and age			
Recipient male sex at birth N (%)	72 (69.9%)	144 (69.2%)	216 (69.5%)
Age at baseline (years) mean ± (S.D.)	51.8 (14.8)	57.5 (13.6)	55.6 (14.2)
Age at 1st kidney transplant (years) mean ± (S.D.)	48.0 (15.0)	54.2 (13.7)	52.1 (14.4)
Recipient race, n (%)			
Caucasian	42 (40.8%)	122 (58.7%)	164 (52.7%)
Hispanic or Latino	2 (1.9%)	1 (0.5%)	3 (1.0%)
Indigenous	0 (0.0%)	4 (1.9%)	4 (1.3%)
Asian	6 (5.8%)	7 (3.4%)	13 (4.2%)
Other	1 (1.0%)	0 (0.0%)	1 (0.3%)
Undisclosed	52 (50.5%)	71 (34.1%)	123 (39.5%)
Proximity to transplant centre, n (%)			
< 25 Km	24 (23.3%)	75 (36.1%)	99 (31.8%)
25–50 Km	26 (25.2%)	34 (16.3%)	60 (19.3%)
51–100 Km	24 (23.3%)	32 (15.4%)	56 (18.0%)
101–200 Km	13 (12.6%)	22 (10.6%)	35 (11.3%)
201–400 Km	6 (5.8%)	23 (11.1%)	29 (9.3%)
401–1000 Km	6 (5.8%)	16 (7.7%)	22 (7.1%)
> 1000 Km	4 (3.9%)	6 (2.9%)	10 (3.2%)
Primary reason for transplant (> 5%), n (%)			
Diabetes	13 (12.6%)	46 (22.1%)	59 (19.0%)
Glomerulonephritis	12 (11.7%)	26 (12.5%)	38 (12.2%)
Polycystic kidney disease	15 (14.6%)	28 (13.5%)	43 (13.8%)
Hypertensive nephrosclerosis	3 (2.9%)	16 (7.7%)	19 (6.1%)
IgA nephropathy	25 (24.3%)	14 (6.7%)	39 (12.5%)
Other defined reasons	24 (23.3%)	57 (27.4%)	81 (26.0%)
HLA match, n (%)			
Fully Matched	6 (5.8%)	1 (0.5%)	7 (2.3%)
Mismatched	97 (94.2%)	207 (99.5%)	304 (97.7%)
Mismatch location, n (%)			
HLA-A	64 (62.1%)	153 (73.6%)	217 (69.8%)
HLA-B	69 (67.0%)	160 (76.9%)	229 (73.6%)
HLA-C	51 (49.5%)	117 (56.3%)	168 (54.0%)
HLA-DP	25 (24.3%)	80 (38.5%)	105 (33.8%)
HLA-DQ	54 (52.4%)	122 (58.7%)	176 (56.6%)
HLA-DR	70 (68.0%)	148 (71.2%)	218 (70.1%)
Other	16 (15.5%)	33 (15.9%)	49 (15.8%)

Participating Canadian transplant centres included: the University of British Columbia, Vancouver, Canada; Western University, London, Canada; McMaster University, Hamilton, Canada; McGill University, Montreal, Canada; the University of Toronto, Toronto, Canada; Dalhousie University, Halifax, Canada; and the University of Alberta, Edmonton, Canada.

The index date for each participant was the date of kidney transplant (Day 0) and data were recorded for the first year following transplantation. Primary data elements included, but were not limited to, patient demographics (age, age at time of first kidney transplant, race, proximity to transplant center); donor source (living/deceased), HLA matching; clinical therapies including induction regimen; CMV infection, syndrome, or disease; the incidence and management of CMV antiviral-related myelotoxicities; and healthcare resource use. Secondary data elements included time to CMV infection, viremia levels, viral load kinetics; event history (phlebitis, anemia, thrombocytopenia, pancytopenia, neutropenia, direct renal tubular toxicity, crystalline nephropathy, leukopenia, other); non-CMV opportunistic infections; graft outcomes; utilization patterns of CMV therapy; and incidence and management of other CMV antiviral-related toxicities.

### CMV prophylaxis and treatment

The following characteristics were extracted from the patient charts to describe antiviral prophylaxis and treatment for CMV: whether anti-viral prophylaxis treatment was prescribed; the stage of use (primary [immediate post-transplant] or secondary [post-primary infection]); and the type of antiviral medication including the dose, unit; frequency and start and stop dates. The response of CMV infection to antiviral treatment was measured using the following data obtained from the patient chart: the date of the first episode of CMV infection; the duration of the episode (days); any change in treatment; (immunosuppression, antiviral prophylaxis, G-CSF, other); whether resistant or refractory CMV Infection or disease occurred (yes/no); and whether resistant infection or disease was confirmed (yes/no).

Myelotoxicity (determined as hematological values below the lower level of normal for each institution) and other adverse consequences of antiviral treatment were recorded using measures obtained from the patient chart. These included: whether CMV antiviral related myelotoxicities occurred (yes/no); the type of myelotoxicity (neutropenia, leukopenia, thrombocytopenia, pancytopenia); other antiviral toxicity recorded (nephrotoxicity, neurotoxicity, electrolyte disturbance, other); grading of the severity (using the NCI-CTCAE Grade 1 - 5) (29); whether toxicity was drug related (yes/no) and if so the drug name (ganciclovir, foscarnet sodium, immunoglobulin, valganciclovir, other); and any change in treatment (immunosuppression, antiviral prophylaxis, G-CSF, other).

### Outcomes and assessments

CMV infection was measured as: (a) the incidence of the first and of each subsequent CMV episode post-transplant, including any combination of CMV infection, CMV syndrome and CMV disease; (b) the time in days to start of the first CMV reactivation (infection, syndrome or disease if noted prior to documented infection) and to each subsequent CMV infection post-transplant; and (c) the peak viral load during first and each subsequent CMV infection post-transplant.
CMV syndrome was defined as viremia accompanied by the presence of classical features of CMV infection including fever, malaise, leukopenia, thrombocytopenia, elevated hepatic transaminases and greater than or equal to 5% atypical lymphocytes (30).
CMV end-organ disease was defined as viremia, which was accompanied by gastrointestinal disease, pneumonitis, hepatitis, nephritis, myocarditis, pancreatitis, encephalitis, retinitis, pulmonary or other classical features of organ involvement (30).
Unmet needs of current prophylaxis were measured as: (a) the incidence of CMV prophylaxis, the descriptions of medications used and the duration of therapy; (b) the incidence of CMV treatment for CMV infection, the description of medications used and the duration of therapy; (c) the incidence and management of CMV antiviral related myelotoxicities and patient outcome associated with neutropenia/leukopenia and (d) the incidence and management of other CMV antiviral-related toxicities (e.g., nephrotoxicity).
Health economic burden was measured as; (a) the incidence and length of stay of all cause hospitalizations, ICU admissions, hospitalization for CMV infection, and hospitalizations associated with neutropenia/leukopenia and their consequences; (b) the number of health care visits post-transplant with a general practitioner, nephrologist and/or urologist; (c) the frequency of CMV surveillance post-transplant (e.g. PCR tests, in-person visits); (d) the number of re-admissions for CMV related morbidity; (e) CMV antiviral therapy and duration of treatment; (f) the incidence and use of Granulocyte Colony Stimulating Factor (GCSF); and (g) the proximity of the patient to the primary transplant hospital to assess feasibility of in-person follow-up visits.
Graft outcome and patient survival were assessed from the incidence and type of graft rejection, graft dysfunction and graft loss based on normal clinical, laboratory and histological parameters. Patient survival was measured using the following data from the patient chart: whether the patient was alive at end of follow up period (yes/no); if no, the date of patient death; and the primary cause of death (i.e. CMV disease, related to CMV including documented CMV infection at death, or other complications relating to the patients transplant surgery, cardiovascular event, other events, or unknown).

### Statistical analysis

All analyses were performed based on the Eligible Population (ELIG) which was a subset of all enrolled participants with complete data available for 365 days, or to the point of death or graft loss. Descriptive statistics were produced for all key study variables for the overall study population. Demographic data included baseline recipient characteristics. Therapeutic data included immunosuppressive therapy, anti-viral prophylaxis and treatment, and other principal therapies that could influence outcome or disease prevalence. Clinical and laboratory data included CMV Ig status at the time of transplant, quantitative viremia, CMV syndrome and disease, graft and participant survival and other outcome variables of interest.

Continuous variables were summarized using the number of non-missing observations, mean, standard deviation (SD), median, minimum, and maximum values and interquartile ranges. Categorical variables were summarized using the number and percentage of participants belonging to each category. Event rates (summarized as the number of participants experiencing that event) and incidence rates (summarized as the total number of events in a group divided by the time at risk), were summarized within the study population and sub- populations of interest for all the dependent variables (e.g. CMV viremia, CMV resistance, developing refractory CMV). Time to event endpoints were calculated using the Kaplan-Meier estimator, and differences between groups were evaluated using the log-rank test. Analysis was performed using SAS version 9.4.

### Ethical approval

This study including all relevant documentation was approved by the Research Ethics Boards at each of the participating sites.

## Results

### Study population

A total of 311 consecutive CMV D+/R- kidney recipients within the index period were enrolled across seven Canadian centers (**Table 1**). There were no screen failures or exclusions. Of these, 103 received a transplant from a living donor (LD) and 208 from a deceased donor (DD); the median age (IQR) at baseline was 58 (46-67) years and at the first kidney transplant was 55 (42, 64) years; and 69.5% were male and 52.7% were Caucasian. Most participants (68%) lived within 100 km of their transplant center. Primary reasons for kidney failure were diabetes (19.0%), polycystic kidney disease (13.8%), IgA nephropathy (12.5%), other glomerulonephritis (12.2%), or for reasons not specifically defined (26.0%). Most grafts were HLA mismatched (97.7%) with typically two mismatches at HLA-A (44.5%), HLA-B (60.1%), HLA-C (45.4%), HLA-DR (49.6%), HLA-DQ (53.5%) and HLA-DP (47.2%) for those reporting these values. No clinically meaningful differences between those receiving an LD or DD graft were observed with regards to participant baseline characteristics.

### Immunosuppression and antiviral prophylaxis

Most patients received induction with basiliximab (61.4%), ATG (24.4%) or another agent (14.1%), principally alemtuzumab, and 93.9% received mycophenolate mofetil (MMF) or mycophenolic acid (MPA) as maintenance immunosuppression, 87.1% received tacrolimus and 77.2% prednisone (**Table 2**). Most participants (62.4%) received all 3 maintenance agents, while 33.4% received two and 4.2% only one. All participants received antiviral CMV prophylaxis, principally with valganciclovir (293, 90.2%), started a median of 1 day post-transplant and lasting for a median of 180 days (range: 2–465 days). Most patients received only one course of antiviral therapy (76.2%), though almost one quarter received two (19.0%) or more courses (4.8%). The duration of each course is shown in **Table 2**. There were no clinically meaningful differences between LD and DD recipients with regards to antiviral prophylaxis. Less than 2% of participants had documented toxicities related to antiviral therapies, of which 28.6% were related to valganciclovir, 14.3% to ganciclovir and 57.1% to other agents (tacrolimus) with antivirals. Outside of myelotoxicity (discussed below), the most common toxicities were neurotoxicity, nephrotoxicity, electrolyte disturbances and other toxicities that were mild (grade 1 – 2). No participants experienced symptoms related to G-CSF administration.

**Table 2:** Immune suppression and antiviral prophylaxis.

	Living Donor (N=103)	Deceased Donor (N=208)	Overall (N=311)
Induction immunosuppression, n (%)			
Anti-human thymocyte immunoglobulin (ATG)	18 (17.5%)	58 (27.9%)	76 (24.4%)
Basiliximab/IL-2 receptor antagonists	76 (73.8%)	115 (55.3%)	191 (61.4%)
Other	9 (8.7%)	35 (16.8%)	44 (14.1%)
Maintenance immunosuppression, n (%)			
Tacrolimus	92 (89.3%)	179 (86.1%)	271 (87.1%)
MMF	96 (93.2%)	196 (94.2%)	292 (93.9%)
Prednisone	80 (77.7%)	160 (76.9%)	240 (77.2%)
N/A	0 (0.0%)	0 (0.0%)	0 (0.0%)
Antiviral prophylaxis, n (%)			
Yes	103 (100.0%)	208 (100.0%)	311 (100.0%)
No	0 (0.0%)	0 (0.0%)	0 (0.0%)
Antiviral used for 1st course of prophylaxis, n (%)			
Valacyclovir	5 (4.6%)	13 (6.0%)	18 (5.5%)
Ganciclovir	5 (4.6%)	9 (4.1%)	14 (4.3%)
Valganciclovir	98 (90.7%)	195 (89.9%)	293 (90.2%)
Courses of antiviral treatment, n (%)			
1 course	81 (78.6%)	156 (75.0%)	237 (76.2%)
2 courses	18 (17.5%)	41 (19.7%)	59 (19.0%)
3 or more courses	4 (3.9%)	11 (5.3%)	15 (4.8%)
Duration of antiviral therapy (Days)*			
**First course, n (patients)**	101	200	301
Mean	173.0	159.7	164.1
SD	63.16	70.97	68.63
Min	63	2	2
Median	182	177	180
Max	435	465	465
**Second course, n (patients)**	17	46	63
Mean	57.8	70.8	67.3
SD	33.45	53.93	49.34
Min	20	4	4
Median	49	56	54
Max	132	243	243
**Third course or greater, n (patients)**	3	8	11
Mean	30.0	50.0	44.5
SD	10.82	32.27	28.97
Min	21	16	16
Median	27	43	36
Max	42	115	115

### CMV infections

**Figure 1:**
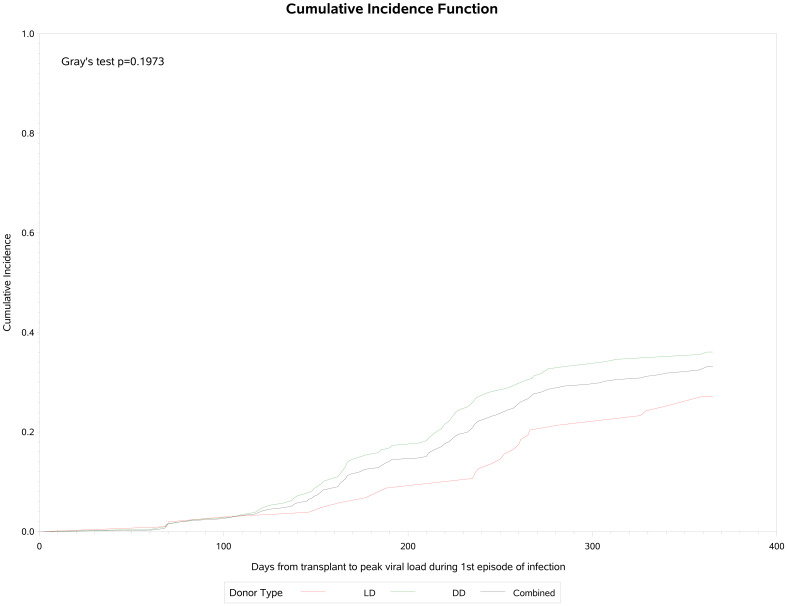
Cumulative incidence of CMV infection following renal transplantation.

A total of 106 (34.1%) participants developed post-transplant CMV infection, of whom 15 (14.2%) (had two and 12 (11.3%) had three or more episodes of infection (**Table 3**). Of these 106 patients, 47 (44.3%) had asymptomatic viremia during follow-up while 59 (55.6%) had CMV disease presenting principally with fever (21.9%), malaise (33.3%), leukopenia (28%) and transaminitis (12%). CMV infection occurred approximately 90 days post-transplant and increased in frequency to a plateau at the end of the first year (**Figure 1**). Breakthrough CMV infection occurred during prophylaxis in approximately 23% of these patients for whom precise dates were available (n=95). CMV disease was diagnosed a median of 223 days (IQR 165, 257) post-transplant, lasted for a median of 34 days (IQR 23, 41)and resolved by 261 days (IQR 192, 306) days post-transplant. Only 7 of these patients (11.9%) had end-organ disease, manifested principally by gastrointestinal or neurological symptoms. CMV infection was slightly more common in DD than LD recipients (viremia: 36.5% *vs* 29.1%, symptomatic: 21.2% *vs* 14.6%) but there was no clinically meaningful or statistically significant difference between LD and DD recipients. Peak viral load during the first infection occurred at a median of 218 days (IQR: 154, 260) days post-transplant with a median value of 14,224 IU/ml (IQR: 3,900, 127,050 IU/ml). Peak viral loads measured in subsequent viremic episodes were later (up to 352 days) and lower (maximum 32,007 IU/ml). Eighty-one patients received a second course of antiviral treatment, including 69 (85.2%) with valganciclovir and 9 (11.1%) with ganciclovir.

**Table 3:** Frequency, timing and clinical category of CMV infection following renal transplantation.

	Living donor (N=103)	Deceased donor (N=208)	Overall (N=311)
Patient with CMV infection, n (%)			
Yes	30 (29.1%)	76 (36.5%)	106 (34.1%)
No	73 (70.9%)	132 (63.5%)	205 (65.9%)
Category of CMV infection, n (%)			
CMV disease	15 (14.6%)	44 (21.2%)	59 (19.0%)
Asymptomatic viremia	15 (14.6%)	32 (15.4%)	47 (15.1%)
Patient episodes of CMV infection, n (%)			
1 episode	22 (73.3%)	57 (75.0%)	79 (74.5%)
2 episodes	6 (20.0%)	9 (11.8%)	15 (14.2%)
3 or more episodes	2 (6.7%)	10 (13.2%)	12 (11.3%)
Transplant to peak viral load (Days)			
N	28	75	103
Q1	183	151	154
Median	248	210	218
Q3	274	243	260
95% CI	(202, 263)	(183, 214)	(194, 222)
Peak viral load during 1st CMV infection (IU/ml)			
Q1	2,025	4,450	3,900
Median	10,300	18,300	14,224
Q3	25,000	152,379	127,050
95% CI	(0, 45956804)	(0, 737366)	(0, 12478139)

### Myelotoxicities

**Figure 2:**
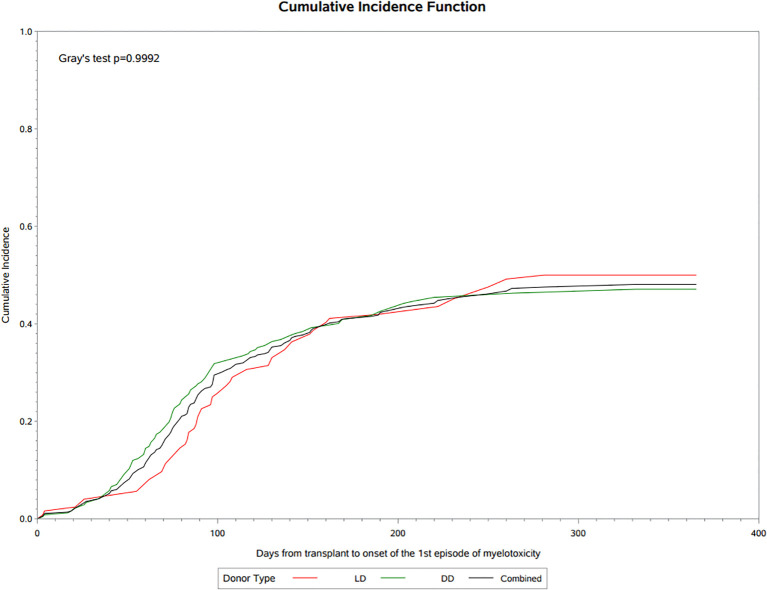
Cumulative incidence of myelotoxicity following transplantation.

A total of 121 (38.9%) patients experienced antiviral related myelotoxicities classified principally as leukopenia or neutropenia, with a frequency that was similar in LD (39.8%) and DD (38.5%) recipients. Cumulative incidence analysis showed that myelotoxicity was observed within 30 days post-transplant, rising rapidly during the first 3 months to 34.5% at day 100 and 46.8% by the end of the first year of follow-up (**Figure 2**). The median onset was 90 (IQR 65, 133) days post-transplant, median duration was 30 (IQR 14, 74) days and only 6 patients (4.9%) had an NCI-CTCAE severity grade above 3. Immunosuppressive therapy was reduced in almost 80% of the patients with myelotoxicity, being decreased on 65(53.7%) or discontinued or interrupted on 43 (35.1%) of the 134 changes recorded, and antiviral therapy was similarly decreased or discontinued in 34 of the 50 (68%) patients on treatment at the time of myelotoxicity. Other agents such as trimethoprim-sulfamethoxazole were discontinued in 9.1% of patients and G-CSF was implemented in 22 (18.2%) patients during the episode of myelotoxicity. A second episode of myelotoxicity occurred in 14 (11.5%) of these patients at a median of 136 (IQR 89, 167) days, with a duration of 39 (IQR 24, 46) days, of which none had an NCI-CTCAE severity over grade 3. Immunosuppressive therapy was decreased, interrupted or discontinued in 94.5%, antiviral therapy discontinued or interrupted in 28.6% and G-CSF was started in 14.3% of these patients. A third or greater episode of myelotoxicity occurred in 5 (4.1%) of patients a median of 174 (IQR 161, 247) days post-transplant, lasting for 70 (IQR 60,80) days, in which no events were above Grade 2 severity. Immunosuppression was decreased or discontinued/interrupted in 2 (40%) patients, and antiviral prophylaxis discontinued or interrupted in 1 (20%) patient. No patients received G-CSF.

### Opportunistic infections

**Figure 3:**
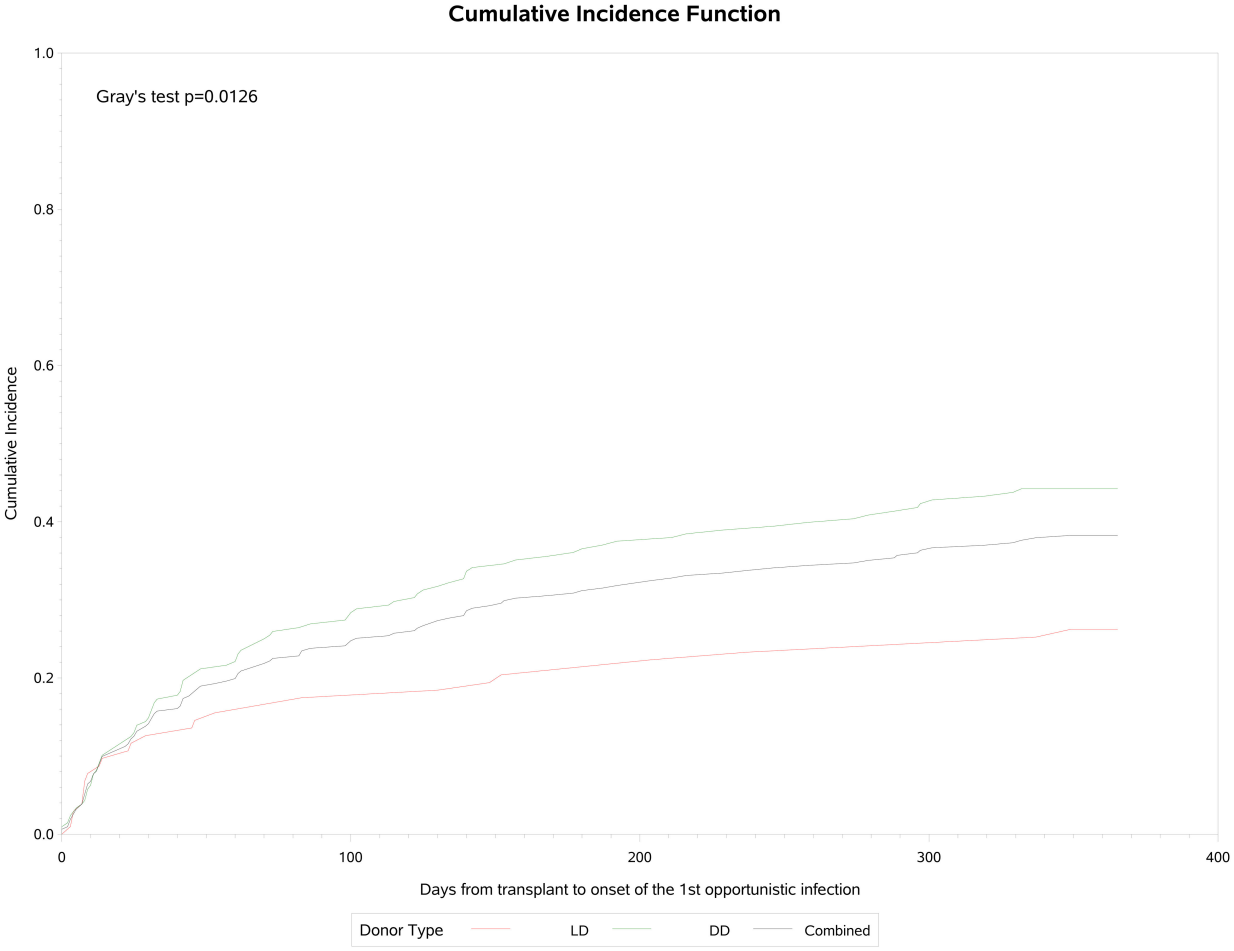
Cumulative incidence of opportunistic infection following transplantation.

A total of 119 (38.3%) patients experienced opportunistic infections post-transplant, which were significantly more common in DD (44.2%) than LD (26.2%) recipients (p=0.002) (**Table 4**). Cumulative incidence analysis (**Figure 3**) showed that infections were first observed within 30 days post-transplant, rising to an inflection at 60–90 days and increasing slowly thereafter to approximately 38% by the end of the first year, with a median onset of 53 (IQR: 14, 142) days post-transplant and a median duration of 28 (IQR: 15, 62). Recurrent opportunistic infections were recorded in 45 (14.5%) of these patients, of whom 15 (4.8%) had three or more episodes. Median onset for the second and third or more episodes was 137 (IQR 77, 213) days and 181 (IQR 109, 236) days respectively, with a duration of 22 (IQR 14, 49) days and 29 (IQR 18, 50) days. Of the 179 total episodes of opportunistic infection reported, 98 (54.7%) were bacterial, 56 (31.3%) were non-CMV viral infections and 30 (16.7%) were fungal in origin. Almost all (97.8%) were Grade 1–3 in severity.

**Table 4:** Opportunistic infection following transplantation by donor source.

	Living donor (N=103)	Deceased donor (N=208)	Overall (N=311)
Patients with opportunistic infections, n(%)			
Yes	27 (26.2%)	92 (44.2%)	119 (38.3%)
No	76 (73.8%)	116 (55.8%)	192 (61.7%)
Patients with 1 or more opportunistic infections, n(%)			
1 infection	16 (59.3%)	58 (63.0%)	74 (62.2%)
2 infections	9 (33.3%)	21 (22.8%)	30 (25.2%)
3 or more infections	2 (7.4%)	13 (14.1%)	15 (12.6%)
Type of 1st infection, n(%)			
Bacterial	8 (29.6%)	49 (53.3%)	57 (47.9%)
Fungal	12 (44.4%)	12 (13.0%)	24 (20.2%)
Viral (not CMV)	7 (25.9%)	31 (33.7%)	38 (31.9%)
Grade of 1st infection, n(%)			
Grade 1	7 (25.9%)	26 (28.3%)	33 (27.7%)
Grade 2	17 (63.0%)	46 (50.0%)	63 (52.9%)
Grade 3	2 (7.4%)	18 (19.6%)	20 (16.8%)
Grade 4	1 (3.7%)	0 (0.0%)	1 (0.8%)
Grade 5	0 (0.0%)	2 (2.2%)	2 (1.7%)
Time from transplant to onset of the 1st infection (Days)			
Mean	93.1	94.0	93.8
SD	109.11	93.89	97.06
Q1	8	19	14
Median	45	61	53
Q3	152	140	142
Duration of the 1st infection (Days)			
Mean	47.4	63.3	59.5
SD	62.94	87.85	82.60
Q1	21	15	15
Median	29	28	28
Q3	36	67	62

As shown in **Table 5**, there were no significant differences in the incidence, number of episodes, causal agents or severity of opportunistic infections between patients who did, or did not, experience post-transplant CMV infection (p=NS), Opportunistic infection occurred significantly later in patients with CMV infection (median: 86, IQR: 20, 180 days, p=0.037) than in those without CMV infection (median: 43, IQR: 14, 127 days) though there was no difference in duration of opportunistic infections between the two groups (median: 28 days).

**Table 5:** Opportunistic infection following transplantation by CMV infection.

	CMV infection (N=106)	No CMV infection (N=205)	Overall (N=311)
Patients with opportunistic infections, n(%)			
Yes	43 (40.6%)	76 (37.1%)	119 (38.3%)
No	63 (59.4%)	129 (62.9%)	192 (61.7%)
Patients with 1 or more opportunistic infections, n(%)			
1 infection	26 (60.5%)	48 (63.2%)	74 (62.2%)
2 infections	9 (20.9%)	21 (27.6%)	30 (25.2%)
3 or more infections	8 (18.6%)	7 (9.2%)	15 (12.6%)
Type of 1st infection, n(%)			
Bacterial	25 (58.1%)	32 (42.1%)	57 (47.9%)
Fungal	6 (14.0%)	18 (23.7%)	24 (20.2%)
Viral (not CMV)	12 (27.9%)	26 (33.2%)	38 (31.9%)
Grade of 1st infection, n(%)			
Grade 1	11 (25.6%)	22 (28.9%)	33 (27.7%)
Grade 2	22 (51.2%)	41 (53.9%)	63 (52.9%)
Grade 3	8 (18.6%)	12 (15.8%)	20 (16.8%)
Grade 4	1 (2.3%)	0 (0.0%)	1 (0.8%)
Grade 5	1 (2.3%)	1 (1.3%)	2 (1.7%)
Time from transplant to onset of the 1st infection (Days)			
Mean	109.4	85.0	93.8
SD	97.48	96.34	97.06
Q1	20	14	14
Median	86	43	53
Q3	180	127	142
Duration of the 1st infection (Days)			
Mean	60.4	59.0	59.5
SD	74.58	87.37	82.60
Q1	16	14	15
Median	29	28	28
Q3	68	61	62

### Hospitalization

**Figure 4:**
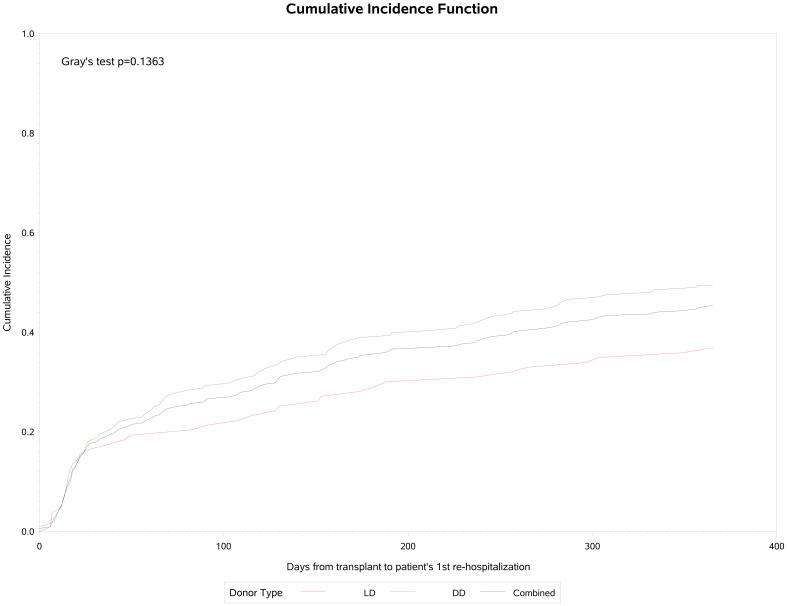
Cumulative incidence of re-hospitalization following transplantation.

A total of 141 (45.3%) participants were re-hospitalized post-transplantation, 103 (36.9%) in the living donor and 38 (49.5%) in the deceased donor group. Of these admissions, 25 (17.7%) were related to CMV infection or leukopenia/neutropenia. Cumulative incidence analysis (**Figure 4**) showed that re-hospitalization commenced early, rising to 28% by day 30 and continuing to 45% by the end of the first year of observation. The median time to first re-hospitalization was 58 (IQR: 18, 158) days and the median duration was 5 (IQR: 2, 11) days. Of these patients, 9 (6.3%) required admission to ICU, for a median of 14 (IQR: 5, 14) days. Fifty (35,4%) of these 141 patients were re-hospitalized a second time, of which 6 (12%) were related to CMV infection or leukopenia/neutropenia. The median time to re-admission was 114 (IQR: 49, 205) days, and median duration was 6 (IQR: 4, 10) days. Two patients (4%) were admitted to ICU for a median of 2 days. And 31 (21.9%) patients were re-hospitalized 3 or more times of which 3 (9.6%) were for CMV infection or neutropenia. The third admission was a median of 145 (IQR: 95, 249) days post-transplant, lasting for a median of 7 (IQR: 3, 12) days and 2 were admitted to ICU for a median of 3 days.

As shown in **Table 6**, the proportion of patients who were re-hospitalized did not differ significantly between patients with or without CMV infection post-transplant (p=NS), But re-hospitalization occurred substantially later in patients with CMV infection (median: 122, IQR: 21, 236 days, p<0.0001) than in those without CMV infection (median: 37, IQR: 16, 130 days) but there was no difference in duration of hospital stay between the two groups (median: 5 days, p=NS).

**Table 6:** Re-hospitalization following transplantation by CMV infection.

	CMV infection (N=106)	No CMV infection (N=205)	Overall (N=311)
Patients re-hospitalized after transplant, n(%)			
Yes	51 (48.1%)	90 (43.9%)	141 (45.3%)
No	55 (51.9%)	115 (56.1%)	170 (54.7%)
Patients with 1 or more re-hospitalizations, n(%)			
1 re-hospitalization	33 (64.7%)	58 (64.4%)	91 (64.5%)
2 re-hospitalizations	12 (23.5%)	19 (21.1%)	31 (22.0%)
3 or more re-hospitalizations	6 (11.8%)	13 (14.4%)	19 (13.5%)
Re-hospitalization related to CMV, n(%)			
Yes	21 (41.2%)	1 (1.1%)	22 (15.6%)
No	30 (58.8%)	89 (98.9%)	119 (84.4%)
Time from transplant to the 1st re-hospitalization (Days)			
Mean	139.8	82.5	103.2
SD	109.70	92.95	102.74
Q1	21	16	18
Median	122	37	61
Q3	236	130	161
Duration of the 1^st^ re-hospitalization (Days)			
Mean	12.7	8.2	9.8
SD	22.97	11.01	16.45
Q1	3	2	2
Median	6	5	5
Q3	14	10	11

In the year following transplantation, all participants (100%) saw a nephrologist (total 5,285 visits, mean 17.0), 82.6% a urologist (total 425 visits, mean 1.4), 57.7% another healthcare provider (total 866 visits, mean 2.8), 39.9% an ambulatory nurse (total 1,293, mean 4.1) and 20.1% a general practitioner (total 162, mean 0.5). There were no clinically meaningful differences between LD or DD recipients, with regards to health care visits.

### Graft and patient survival

**Figure 5:**
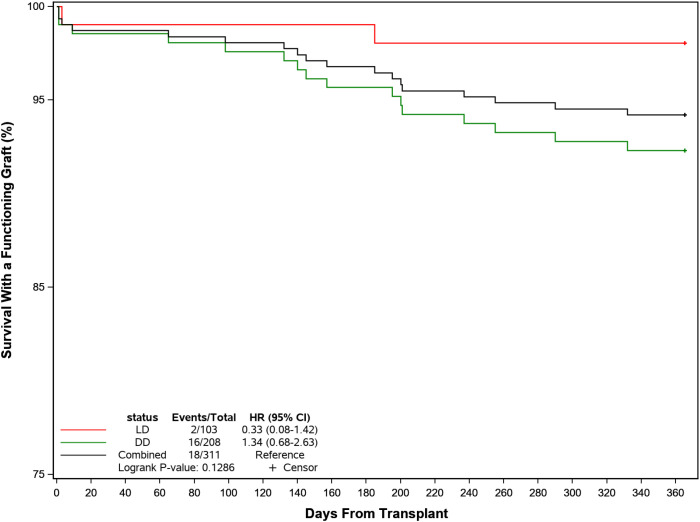
Probability of recipient survival with a functioning kidney graft following transplant by donor source.

Biopsy-proven acute graft rejection (BPAR) occurred in 20 patients (6.4%) overall (LD 4.9% and DD 7.2%, p=NS) after a median of 107 (IQR: 36, 202) days. Three of these patients received biologic therapy with rituximab or ATG. Ten patients (3.2%) lost their graft (LD 1.0%, DD 4.3%), all for reasons other than rejection. By 1 year post-transplant, 10 patients (3.2%) had died (LD 1.0%, DD 4.3%). Death occurred at a median of 152 days (IQR: 140, 201) days post-transplant due principally to cardiovascular events (n=5) and other causes (n=3). Only 1 case was reported related to CMV infection. Overall, 293 of the 311 patients (94.2%) were alive with a functioning graft by 1 year (LD 98.1%, DD 92.3%) (**Figure 5**).

**Figure 6:**
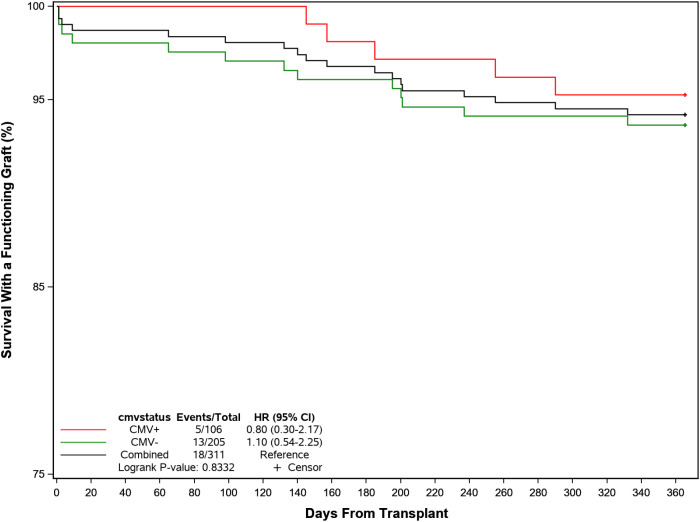
Probability of recipient survival with a functioning kidney graft following transplant by CMV infection.

Graft and patient survival were similar in patients with or without post-transplant CMV infection (**Figure 6**). BPAR was reported in 8 (7.5%) participants with post-transplant CMV infections compared to 12 (5.9%) without, although rejection occurred later in the former at a median of 150 days (IQR: 98, 248) days compared with a median of 64 days (IQR: 14, 199) days in those without infection. Graft loss was higher in patients without CMV infection (4.4% *vs* 0.9%) and 4 participants (3.8%) with CMV and 6 participants (2.9%) without CMV had died by the end of the follow-up period. Overall, 101 (95.3%) and 192 (93.7%) in each group were alive with a functioning graft by 1 year post-transplant. [Post-text Table 14.10.2].

## Discussion

This study provides a detailed and comprehensive understanding of current real-world Canadian practice regarding the management, clinical outcomes and disease burden of patients at the highest risk of CMV infection as a foundation for precision medicine care. It highlights the limitations of current therapy and defines the dynamic patterns of CMV infection in relation to other cardinal events including leukopenia, rejection, infection and hospitalization, elucidating the input parameters to enable the clinical and economic modeling of strategies to optimize therapy and healthcare resource utilization in this complex aspect of transplantation medicine (17, 31–34).

CMV is a ubiquitous pathogen of enormous biological complexity and consequence (35). Primary CMV infection occurs in 40-90% of people globally (36), following which the virus establishes long-term latency by modulating innate and adaptive immunity to facilitate immune evasion (37). Reactivation may occur in the context of host immune dysfunction due to intrinsic disease or external immunosuppression, while reinfection may follow contact with another infected source despite natural immunity (37). By virtue of its frequency, biological complexity and paucity of therapy, CMV has been one of the most challenging pathogens in transplantation (38). It complicates the clinical course through direct effects (including systemic viral infection and end-organ disease) (39), and potentially via indirect consequences including opportunistic bacterial, fungal or other viral diseases, by increasing the risk of rejection and graft loss, or through thrombotic events and accelerated cardiovascular disease (6, 38, 40–42). Current anti-viral treatment has reduced the incidence and severity of CMV disease, although leukopenia and other consequences may decrease the potential benefit (43), so that routine prophylaxis is normally restricted to patients at elevated risk defined by CMV serostatus, increased age and immunocompromised state (44).

Systematic review confirms the benefit of antiviral medication in preventing CMV infection, CMV disease and all-cause death compared with placebo or no treatment (45). Universal prophylaxis and preemptive therapy are the preferred treatment strategies (46), the former offering potentially superior outcomes particularly in high-risk D+/R- subjects with fewer opportunistic infections, rejections and improved graft and patient survival, while the latter offers lower costs and drug exposure and lower rates of late CMV and of viral resistance (47, 48). The limitations of universal prophylaxis are evident in the current study where patients received treatment for a median of 180 days, consistent with current guidelines (8, 18, 25, 26, 49). CMV infection was delayed (to a median of over 200 days) but occurred in 34% of patients of whom over half had CMV disease or end-organ involvement and one quarter had recurrent episodes of infection. Because of these problems, investigators have proposed a hybrid approach in which prophylaxis is followed by long-term pre-emptive monitoring to rapidly identify and treat break-through infection (50, 51). But while appealing, this approach is not without challenge since most patients have returned home by this point, which in the current study was up to 1,000 km from the transplant clinic.

The dosing strategy and duration of CMV treatment are influenced by both costs and adverse effects, of which the most common is leukopenia occurring in 20-80% of patients (43, 52). The incidence, severity and burden depend on a combination of factors including the drug, dose, co-incident therapy, renal function and nucleotide polymorphisms which control drug metabolism and elimination (53). Almost 40% of patients in this study experienced myelotoxicities presenting as leukopenia or neutropenia (54). Myelotoxicity occurred within 30 days post-transplant, rising to almost half of all patients by the end of the first year of follow-up. While leukopenia was rarely serious by NCI-CTCAE severity grade it triggered a reduction or discontinuation of immunosuppression or antiviral therapy in up to 60% of patients increasing the potential for CMV infection, development of resistant strains (55, 56) or graft rejection (57). Systematic review confirms that such reduction in treatment is common in kidney transplantation while, as observed here, the safe and effective alternative of G-CSF is less frequently employed (54, 58). Letermovir, a new antiviral agent which inhibits the CMV viral terminase complex, offers the opportunity to change current practice (59) since antiviral efficacy is comparable to valganciclovir, but leukopenia was reduced by more than half (26% *vs*. 64%) and fewer patients discontinued treatment due to adverse effects (60–62). Caution may be required due to recognized drug interactions with tacrolimus and cyclosporine (61).

Prudent immune suppression, meticulous surgical intervention and improved antimicrobial therapy have reduced the incidence and impact of opportunistic infection and re-hospitalization in kidney transplantation (1, 3, 63). Most infections and hospitalizations reported here occurred in the first 100 days consistent with prior experience. Opportunistic infections occurred in almost 40% of patients, were significantly more common in DD than LD recipients, but were generally mild to moderate in severity with few patients needing ICU admission. Approximately half were bacterial, one third were viral (non-CMV) and 15% were fungal in origin. Almost half of all patients were re-hospitalized post-transplantation, 20% related to leukopenia/neutropenia or CMV infection. Despite these complications, graft and patient outcomes were excellent. Biopsy-proven acute graft rejection (BPAR) occurred in only 6% of patients overall, the majority of these within the first month long preceding CMV infection. This suggests that the broadly reported relationship between infection and BPAR may be consequential rather than causal (17), mediated through the common reduction of IST in the face of leukopenia. By the end of follow-up, 94% of patients remained alive with a functioning graft with only 1 death attributed to CMV infection.

Graft and patient survival were similar in patients with or without post-transplant CMV infection. BPAR was reported in 8 (7.5%) participants with post-transplant CMV infections compared to 12 (5.9%) without, although rejection occurred later in the former at a median of 150 days (range: 36–283 days) compared with a median of 64 days (range: 6–256 days) in those without infection. Graft loss was higher in patients without CMV infection (4.4% *vs* 0.9%) and 4 participants (3.8%) with CMV and 6 participants (2.9%) without CMV had died by the end of the follow-up period. Overall, 101 (95.3%) and 192 (93.7%) in each group were alive with a functioning graft by 1 year post-transplant. We do not yet have definitive explanations for these marginal differences, though are exploring whether they reflect variations in immune suppression and/or viral prophylaxis.

## Limitations and conclusions

This study has limitations including selection bias, information bias and confounding which are inherent to observational design. To minimize selection bias, the study was conducted over a period in which laboratory diagnosis and clinical management of CMV remained constant, from a representative cohort of regional centers performing over 60% of the transplants annually in Canada, all working within the framework of the Canadian Blood Services to ensure national coordination in laboratory and clinical practice (64, 65). While information bias may occur from many sources, stringent efforts were made to reduce these by continuous digital data screening to define data trends and identify missing, extreme and implausible values and by formal data review with the contributing investigators. Nor do we include analysis of cellular immunity, a critical defense mechanism in which a broad range of effector-memory, NK-like CD8 cells and others play an important role in controlling the expression of this virus (26). Exploring the consequences of immune suppression and leukopenia is therefore of particular importance in this aspect.

Within these limits, we may draw the following conclusions. First, CMV infection represents an important and continuing challenge to clinical care, for which current strategies are deficient. Consequently, even within the framework of highly structured and widely approved clinical guidelines, enormous variability occurs in the application of current prevention. Second, the myelotoxicity of current prophylaxis is an important and perhaps under-recognized problem, affecting as it does a high proportion of patients and resulting in reduction in dose of both antiviral agents and immune suppressants. It is feasible that this response contributes to both the persistence and recurrence of CMV infection observed in many patients and the development of acute – and perhaps chronic – rejection in these patients. Finally, the introduction of new agents such as letermovir without appreciable myelotoxicity offers the opportunity to optimize our strategies for care, potentially minimizing the burden of this disease. But the complexities of dose-related pharmacokinetic interactions with key immunosuppressive drugs, and the potential for simply delaying CMV expression require careful consideration to optimize treatment strategies. We anticipate that the granular data obtained in this study will enable detailed modeling to develop the foundation for effective precision medicine strategies.

## Data Availability

These data are confidential clinical information under the custodianship of the regional health authorities and as such are not freely available. Reasonable requests for access may be reviewed by the principal investigator and partners. Requests to access the datasets should be directed to paul.keown@syreon.com.
