# Correction: Clinical management and burden of cytomegalovirus in D+/R-Kidney transplant recipients in Canada

**DOI:** 10.3389/fimmu.2025.1712602

**Published:** 2025-10-14

**Authors:** John Gill, Andrew A. House, Zain Chagla, Jean Tchervenkov, S. Joseph Kim, Amanda Vinson, Carlos Cervera, Paul A. Keown, Sonia Lai Wing Sun, Christina Khoury, Christiane Ghakis

**Affiliations:** ^1^ Department of Medicine, University of British Columbia, Vancouver, BC, Canada; ^2^ Department of Medicine, Western University, London, ON, Canada; ^3^ Department of Medicine, McMaster University, Hamilton, ON, Canada; ^4^ Department of Surgery, McGill University, Montreal, QC, Canada; ^5^ Department of Medicine, University of Toronto, Toronto, ON, Canada; ^6^ Department of Medicine, Dalhousie University, Halifax, NS, Canada; ^7^ Department of Medicine, University of Alberta, Edmonton, AB, Canada; ^8^ Medical Affairs, Merck Canada Inc, Kirkland, QC, Canada

**Keywords:** cytomegalovirus, kidney transplantation, antiviral prophylaxis and treatment, immunosuppression, superinfection, leukopenia, hospitalization, graft failure

There was a mistake in **Figure 2** as published. The X axis legend was incorrect and should read: “Days from transplant to onset of the 1st episode of myelotoxicity”. The corrected **Figure 2** appears below.

**Figure 2:**
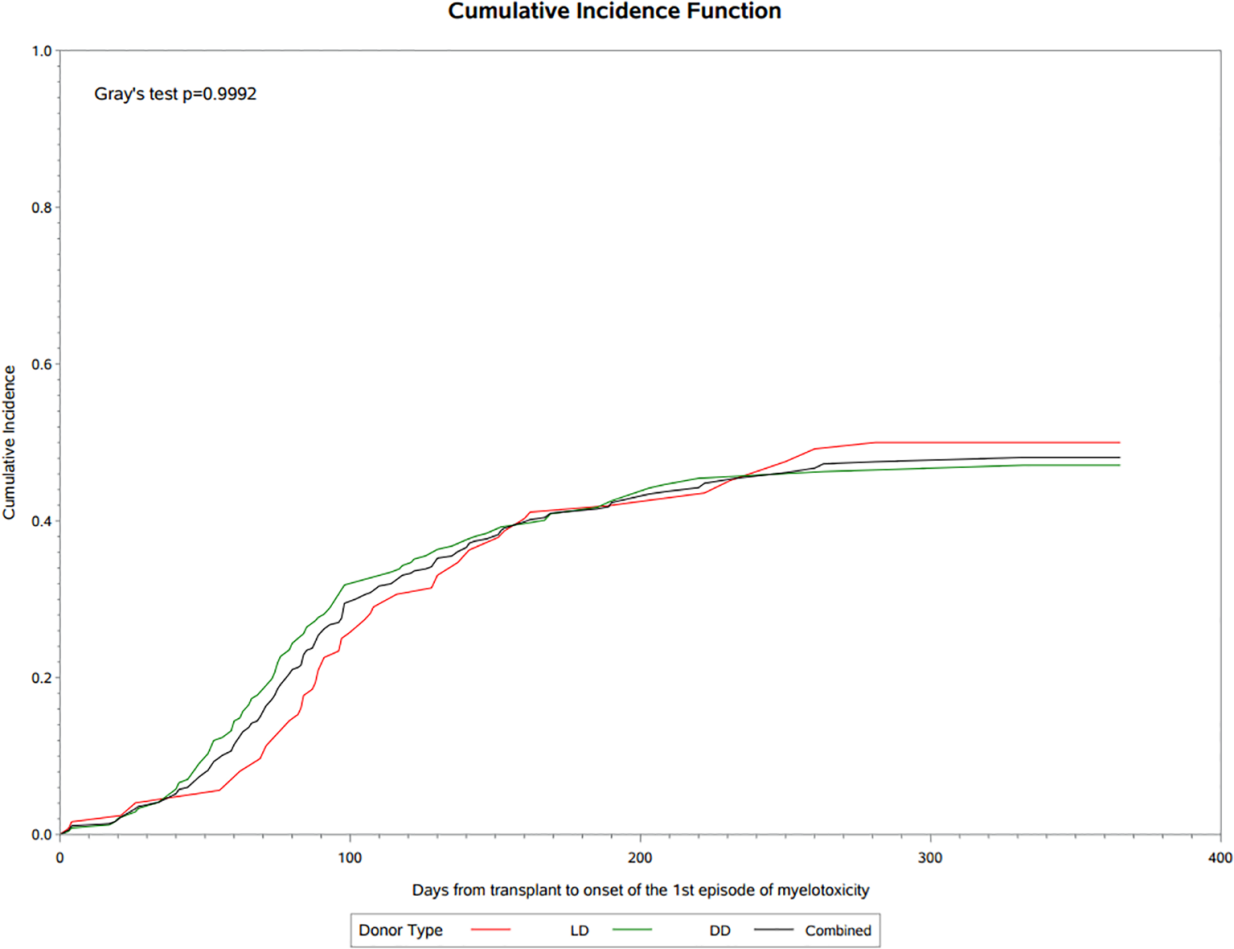
Cumulative incidence of myelotoxicity following transplantation.

The original version of this article has been updated.

